# Chronic Subdural Hematoma: Pathophysiology, Diagnosis, and the Emerging Role of Middle Meningeal Artery Embolization

**DOI:** 10.3390/jcm15114134

**Published:** 2026-05-27

**Authors:** Nikodem Kuczyński, Dawid Pilewski, Edyta Zomkowska, Wojciech Pulka, Mariusz Sowa

**Affiliations:** 1 Faculty of Medicine, Collegium Medicum, Mazovian Academy in Płock, 09-402 Płock, Poland; n.kuczynski@mazowiecka.edu.pl; 2 Faculty of Health Science, Collegium Medicum, Mazovian Academy in Płock, 09-402 Płock, Poland; 3 Department of Neurosurgery, Neurotraumatology and Spinal Surgery, Regional Hospital in Elblag, 82-300 Elblag, Poland; ezomkowska14@gmail.com (E.Z.); ns.wojciechpulka@gmail.com (W.P.); 4 Department of Neurology and Neurosurgery, Faculty of Medicine, Collegium Medicum, University of Warmia and Mazury in Olsztyn, Aleja Warszawska 30, 10-082 Olsztyn, Poland; neurochirurgia@uwm.edu.pl

**Keywords:** chronic subdural hematoma, middle meningeal artery embolization, recurrence, minimally invasive treatment, neurointervention, angiogenesis, inflammation

## Abstract

Chronic subdural hematoma (CSDH) is a common neurological condition, particularly in the elderly, characterized by a complex pathophysiology involving inflammation, angiogenesis, and recurrent microhemorrhages rather than a purely mechanical process. Although surgical evacuation remains the standard treatment, recurrence rates remain considerable, prompting the search for alternative and adjunctive therapies. This narrative review summarizes current evidence on the pathophysiology, diagnostic approaches, and management of CSDH, with particular emphasis on middle meningeal artery embolization (MMAE). A comprehensive literature search of major medical databases, including PubMed, Scopus, and Web of Science, was performed to identify relevant randomized controlled trials (RCTs), observational studies, and meta-analyses. Available evidence suggests that MMAE may reduce recurrence rates and the need for reoperation, particularly when used as an adjunct to surgery. However, results from RCTs remain mixed, and not all studies have demonstrated significant benefit on primary clinical endpoints. While MMAE has emerged as a promising minimally invasive approach targeting the vascular supply of hematoma membranes, further high-quality studies are required to establish standardized indications, optimize procedural techniques, and clarify long-term outcomes and comparative effectiveness.

## 1. Introduction

CSDH is a common and increasingly important neurosurgical condition characterized by the accumulation of blood, fluid, and degradation products between the dura mater and the arachnoid membrane [1]. It typically consists of blood of varying ages enclosed within neomembranes, reflecting its dynamic and evolving nature rather than a static hemorrhagic event [2]. Radiologically, CSDH is most often identified on computed tomography (CT) as a crescent-shaped, hypoattenuating collection that may become heterogeneous over time due to recurrent bleeding or membrane activity [3].

Although traditionally attributed to the rupture of bridging veins following minor head trauma, the pathophysiology of CSDH is now understood to be considerably more complex and multifactorial [4]. Increasing evidence suggests that chronic inflammation, angiogenesis, and repeated microhemorrhages from fragile neovessels within the outer membrane play a central role in hematoma persistence and expansion [5]. This evolving understanding has important therapeutic implications, as it suggests that CSDH may involve not only mechanical bleeding but also ongoing inflammatory and angiogenic processes.

CSDH predominantly affects the elderly population, with incidence rates ranging from 1.72 to 20.6 per 100,000 individuals and rising sharply with age [3]. In patients older than 80 years, the incidence has increased dramatically over recent decades, reaching as high as 129.5 per 100,000 [6]. This trend is expected to continue, driven by population aging, increased use of antithrombotic medications, and higher rates of falls and head trauma [7,8]. Projections suggest that CSDH may become the most common neurosurgical condition in some countries by 2030, with substantial implications for healthcare systems [9]. The associated burden is considerable, including high hospitalization rates, significant healthcare costs, and a growing demand for surgical and interventional management [10].

Clinically, patients with CSDH present with a wide spectrum of symptoms, including headache, confusion, gait disturbances, and reduced levels of consciousness, although presentations can vary widely [1,11]. Seizures may occur in a subset of patients, and the clinical course is often insidious, developing over weeks following the initial insult [11]. Importantly, recurrence remains a major clinical challenge, with rates reported between 0% and 76%, and reoperation required in approximately 10–20% of cases [12]. Risk factors for recurrence include advanced age, anticoagulant use, bilateral hematomas, and unfavorable hematoma characteristics such as thick membranes or large volume [7,8].

The standard management of symptomatic CSDH has historically relied on surgical evacuation, most commonly via burr-hole craniostomy, which effectively relieves mass effect and improves neurological symptoms [5,13]. However, surgery does not address the underlying pathophysiological processes driving recurrence, and postoperative complications remain a concern, particularly in elderly patients with multiple comorbidities. In response, alternative and adjunctive treatment strategies—including pharmacological therapies such as statins, corticosteroids, and antifibrinolytics—have been explored, although their efficacy remains variable and in some cases controversial [14].

In this context, increasing attention has been directed toward the vascular supply of the hematoma membranes, particularly the role of the middle meningeal artery (MMA). The MMA, a branch of the external carotid artery (ECA), provides arterial supply to the dura mater and has been identified as a key contributor to the neovascularization of the outer membrane in CSDH [15]. Anatomical variations and anastomoses of the MMA, including connections with the ophthalmic circulation, are of particular clinical relevance, as they influence both the safety and technical considerations of endovascular interventions [16].

Building on this anatomical and pathophysiological understanding, MMAE has emerged as a novel, minimally invasive therapeutic strategy. By targeting the vascular supply of the pathological membrane, MMAE aims to interrupt the cycle of inflammation, angiogenesis, and recurrent bleeding that underlies CSDH progression [5,17]. Initially described in the early 2000s, MMAE has gained increasing clinical acceptance as both an adjunct to surgical evacuation and, in selected cases, a primary treatment modality [18,19].

Recent clinical data, including multicenter observational studies and RCTs, have provided further insights into the safety and efficacy of MMAE in the management of CSDH. As the understanding of both the anatomical basis and biological mechanisms of the disease continues to evolve, integrating endovascular techniques into standard treatment algorithms may offer a targeted approach to reducing recurrence rates in selected patient populations, though its impact on long-term functional outcomes requires further validation.

## 2. Methods

This study was conducted as a narrative review of the current literature on CSDH, with particular focus on pathophysiology, diagnostic approaches, and emerging treatment strategies, including MMAE. A structured literature search was performed using major medical databases, including PubMed, Scopus, and Web of Science, to identify relevant studies published up to March 2026. Search terms included combinations of “chronic subdural hematoma,” “middle meningeal artery embolization,” “pathophysiology,” “imaging,” “diagnosis,” and “treatment.”

Eligible sources included RCTs, observational studies, meta-analyses, systematic reviews, and selected narrative reviews relevant to the scope of this article. We included human studies, predominantly English-language publications; however, three non-English-language articles considered highly relevant to the topic were also included. Animal studies and conference abstracts without available full texts were excluded.

Articles were initially screened based on titles and abstracts, followed by full-text assessment for relevance and scientific quality. In cases of overlapping or conflicting evidence, priority was given to more recent studies, larger cohorts, higher methodological quality, and peer-reviewed publications, particularly those evaluating the efficacy and safety of MMAE. Reference lists of included articles and recent reviews were additionally hand-searched to identify further relevant publications. Two authors independently screened titles/abstracts and subsequently reviewed full texts. Disagreements were resolved by consensus.

Findings were synthesized qualitatively, with emphasis on identifying consistent observations, emerging therapeutic trends, and areas of ongoing controversy in the management of CSDH. Due to the narrative nature of this review, no formal meta-analysis or quantitative synthesis was performed.

## 3. Embryology and Anatomy of the MMA

The MMA is a significant branch of the ECA, predominantly originating from the maxillary artery (MA) and entering the cranial cavity via the foramen spinosum (FS) to supply a substantial portion of the cranial dura mater [15]. Its intricate embryological development leads to numerous variations in both its origin and branching patterns [20]. Various atypical origins of the MMA have been documented, and in some instances, unrecognized anastomoses with other major vessels are present. These vascular connections can serve as either beneficial or potentially hazardous pathways during endovascular procedures [21].

### 3.1. Embryological Development of the MMA

The MMA emerges during early human embryogenesis from the stapedial artery (SA) [22]. Bertulli and Robert [22], Toma [23], Bonasia et al. [15], and Menshawi et al. [24] indicate that the MMA first appears between days 21 and 50 of gestation (approximately 3–7 weeks) and reaches its definitive form by Padget stages III–VI (9–24 mm embryos, around 5–8 weeks). In these accounts, the SA—arising from the dorsal remnant of the second aortic arch—gives off a maxillo-mandibular branch that, following regression of the stapedial vessel, is incorporated into the ECA system via anastomosis with the MA [22].

Variant origins from the internal carotid artery (ICA), ophthalmic artery (OphA), basilar artery (BA), or occipital artery (OA) result from persistence or regression anomalies of these embryonic channels. These embryology-driven variants account for many clinically significant anastomoses relevant in neurosurgical and endovascular procedures [16].

### 3.2. Typical Origin of the MMA

The MMA generally originates from the mandibular segment of the MA, situated between the lateral pterygoid muscle and the sphenomandibular ligament [15]. It is typically the initial and largest branch of the MA and courses between the two roots of the auriculotemporal nerve [25]. In some instances, the MMA shares a common origin with the accessory meningeal artery (aMA), whereas in others it arises independently [26]. Low et al. [27] documented a notable dissection case in which the MMA originated from the distal third of the MA, with the concomitant absence of the FS and a potential fusion of the bony groove of the MMA with the superior orbital fissure.

**Figure 1:**
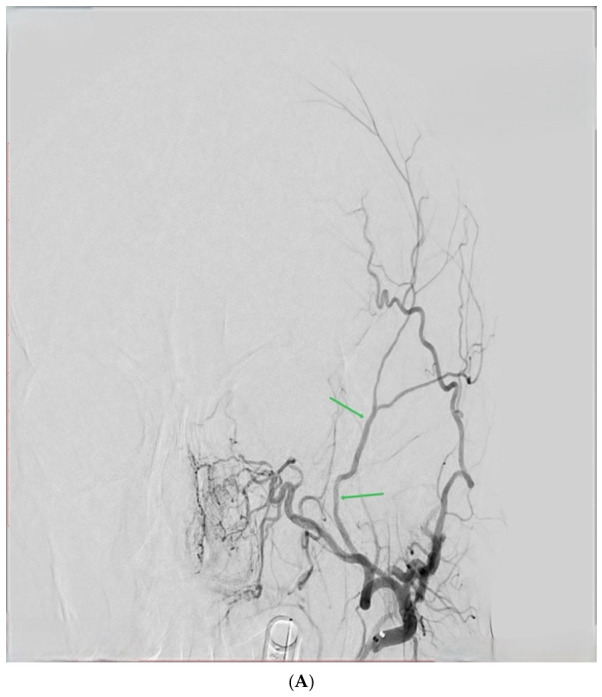

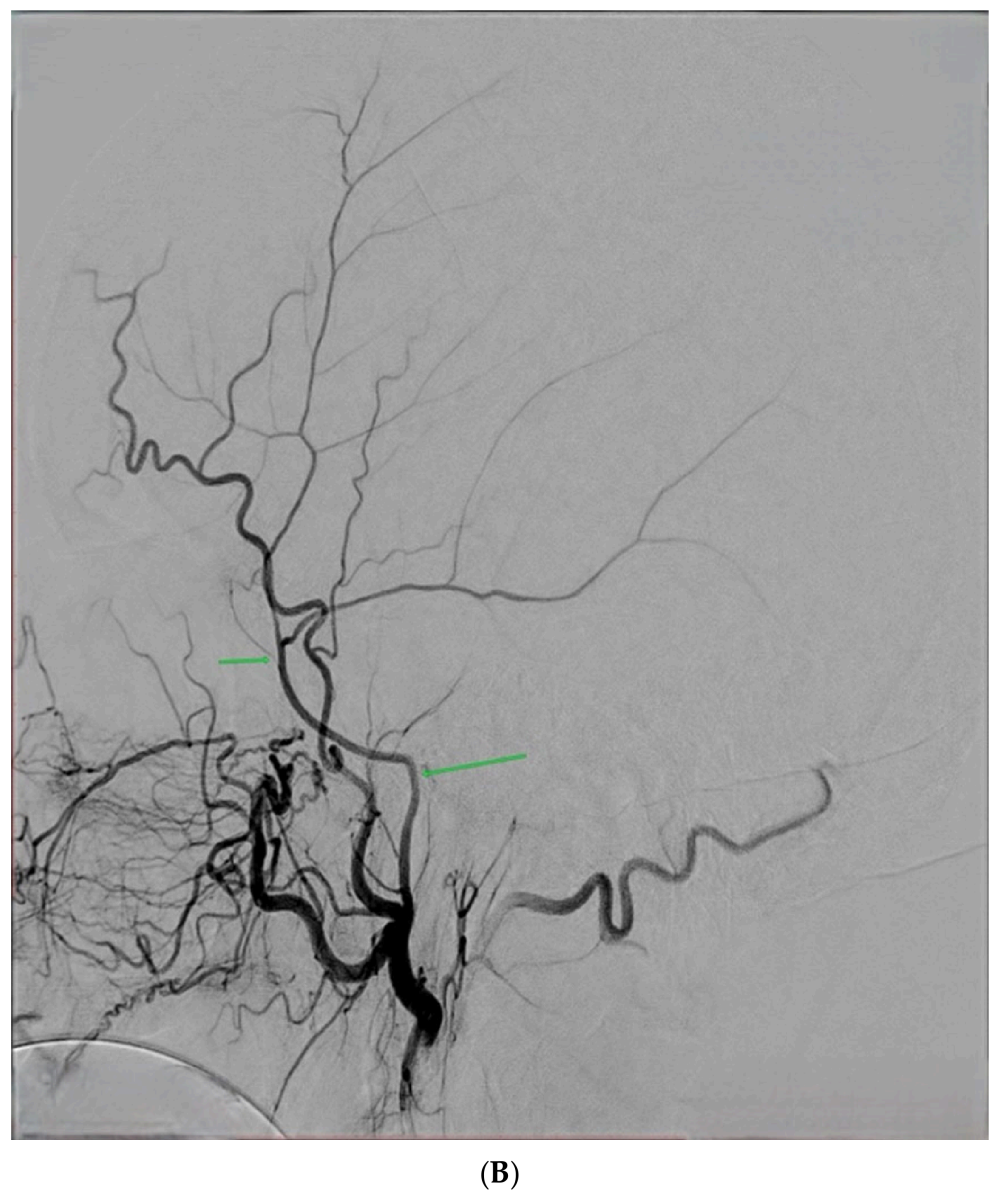
Anterior–posterior projection (**A**) and lateral projection (**B**) of angiography of the left ECA (the typical course of the MMA from the MA is indicated by the green arrow). Own material.

From its origin, the proximal extracranial segment of the MMA extends to its entry into the FS. When the artery arises more anteriorly, this segment follows a more oblique posterior course. At the level of the FS, the vessel bends anteriorly and laterally to follow the temporal fossa, a characteristic feature often observed in digital subtraction angiography (DSA). After entering the skull base and the middle cranial fossa, the artery continues laterally along a groove on the greater wing of the sphenoid bone [15]. A typical MMA angiographic presentation is shown in Figure 1.

### 3.3. Segmentation of the MMA

Detailed angiographic studies have classified the MMA into several distinct segments, each characterized by specific branching patterns and relationships with the surrounding bone and dura mater [28]:

- Extracranial segment: Extends from its origin from the MA to the FS.
- Horizontal segment: Courses anterolaterally within the middle cranial fossa and gives rise to cavernous and petrosal branches.
- Temporal segment: Ascends along the temporal convexity and gives rise to posterior convexity branches.
- Pterional segment: Travels toward the pterion, where it typically divides into anterior and posterior branches.
- Coronal segment: Extends toward the coronal suture, forming rich anastomoses across the midline.

This segmentation framework is particularly useful in endovascular planning, as each segment presents distinct anastomotic risks as well as potential opportunities for targeted embolization.

### 3.4. Dural Territories Supplied by the MMA

The MMA supplies the majority of the supratentorial dura, including the lateral convexities, the falx cerebri, tentorial insertions, and the calvarial bone through transosseous branches. This extensive vascular distribution helps explain why MMA embolization can effectively devascularize the membranes of CSDH, which depend largely on MMA-derived blood supply for their pathological neovasculature [15,29].

## 4. Major Branches and Anastomoses of the MMA

The MMA gives rise to several clinically significant branches that supply the cranial dura mater and establish numerous extracranial–intracranial anastomoses, which are of particular importance during endovascular procedures:

- Anterior branch: Supplies the dura of the frontal and anterior parietal convexities. This branch may reach the midline and anastomose with the anterior falcine branch of the OphA [29,30]. Anastomoses with the lacrimal branch of the OphA through the meningolacrimal artery are of high clinical significance, as reflux during embolization in this region may result in retinal ischemia [16].
- Posterior branch: Supplies the parietotemporal dura and the posterior convexity. It is frequently targeted during embolization for CSDH, as it provides vascular supply to the membranes overlying the parietal convexity [16].
- Petrosal branch: Typically arises from the proximal segment of the MMA shortly after the artery passes through the FS, although it may also originate within or just below the foramen [31]. It courses toward the petrous apex and may anastomose with the internal auditory artery arising from the anterior inferior cerebellar artery. The petrosal branch also gives rise to the superior tympanic branch, which supplies the facial nerve and the geniculate ganglion [16].
- Petrosquamosal branch: Courses along the petrosquamous suture and contributes to the transosseous vascular supply of the posterolateral floor of the middle cranial fossa, the lateral tentorium, and the dura of the superior posterior fossa. It forms anastomoses with the jugular branch of the ascending pharyngeal artery, the medial and lateral tentorial arteries, and the mastoid branch of the occipital artery [16].
- Falcine arteries: Arise from both the anterior and posterior branches of the MMA. These vessels may anastomose with the anterior falcine artery, the ethmoidal branches of the OphA, the anterior cerebral artery, and the posterior meningeal artery [16].
- Cavernous branch: Usually originates from the petrosal branch and supplies the lateral wall of the cavernous sinus [16]. This branch may anastomose with the posterior branch of the inferolateral trunk of the internal carotid artery, creating a potentially hazardous extracranial–intracranial anastomosis and providing vascular supply to the Meckel (trigeminal) cave and its associated nerves [17]. Table 1 summarizes the major branches of the MMA, their vascular territories, anastomoses, and clinical relevance.

Several MMA–orbital anastomotic variants have also been described [17,29,30]. These include the meningo-ophthalmic variant, in which the distal OphA is supplied entirely by the MMA; the meningo-lacrimal variant, characterized by a connection between the sphenoid branch (orbital branches) of the MMA and the lacrimal artery; and variants in which the frontal branch of the MMA reaches the midline and anastomoses with falcine branches of the OphA [29,30].

Recognition of these vascular connections is essential, as embolization of meningeal vessels supplying a lesion may carry a risk of ophthalmic complications if these anastomoses are present. Consequently, careful angiographic identification of the OphA is crucial during diagnostic and therapeutic procedures.

## 5. Variants of the MMA Origin

Variants in the origin of the MMA arise from its complex embryologic development and may create clinically significant collateral pathways [20,21]. Although the MMA most commonly arises from the MA [15], two of the most important alternative origins—due to their implications for endovascular safety—are those from the OphA and the basilar artery (BA).

### 5.1. Origin from the OphA

**Figure 2:**
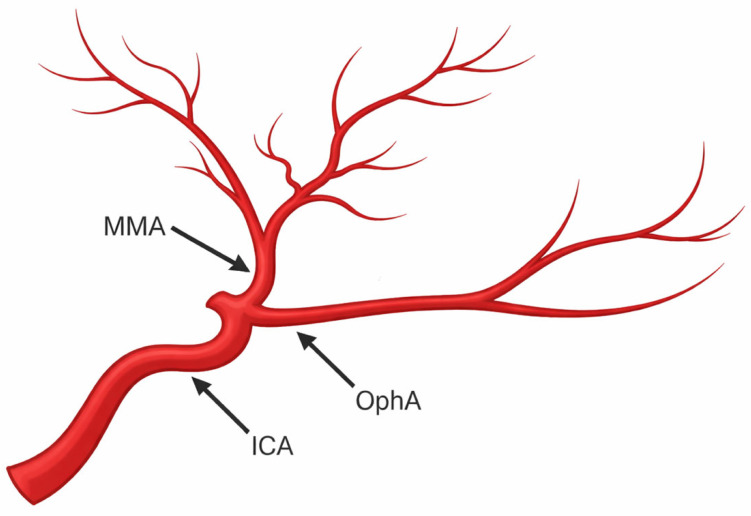
MMA arising from the OphA. Modified from Azab et al. [26].

A rare variant (~0.5%) involves the MMA arising from the OphA instead of the MA (Figure 2) [32,33]. First described by Curnow in 1873 [34], it is frequently associated with absence of the FS and may originate either directly from the OphA or from the lacrimal artery [33,35]. Reported configurations range from complete OphA-derived MMA to cases where only the anterior division arises from it [15,26,36,37]. This variant is clinically relevant due to its direct connection to the ophthalmic circulation and associated risk of retinal ischemia during embolization.

### 5.2. Origin from the BA

**Figure 3:**
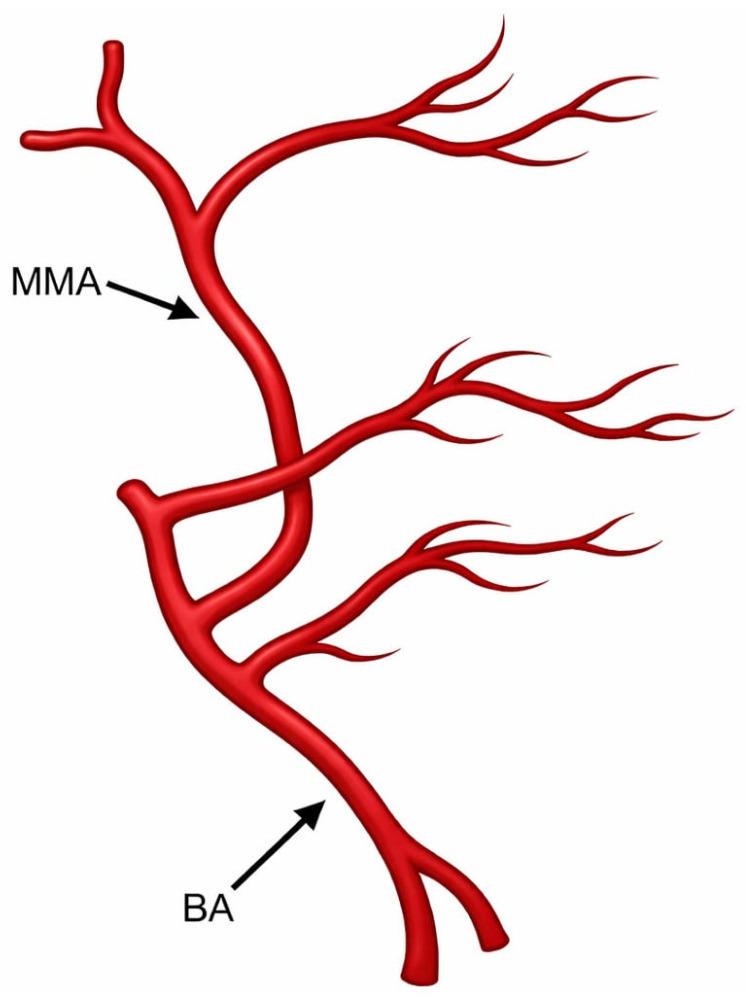
MMA arising from the BA. Modified from Azab et al. [26].

Another rare variant is MMA origin from the BA (Figure 3), first described by Altmann in 1947 [38]. It likely reflects persistence of embryonic trigeminal or gasserian arterial channels [26]. Typically the posterior MMA branch arises from the BA—less commonly the entire trunk—with origins usually between the superior cerebellar artery and anterior inferior cerebellar artery, or occasionally from the posterior inferior cerebellar artery [39,40,41,42]. The FS may be absent or associated with a hypoplastic accessory meningeal artery [39,43]. This configuration is clinically important because it creates direct communication with the posterior circulation, increasing the risk of brainstem or cerebellar embolization.

Understanding these extensive embryological variations is not merely academic; it is directly relevant to clinical outcomes. For instance, an unrecognized origin from the BA drastically increases the risk of catastrophic brainstem or cerebellar embolization during the procedure. Thus, meticulous anatomical mapping is a prerequisite for safe endovascular intervention.

## 6. Pathophysiology of CSDH

### 6.1. Structure of the Dura–Arachnoid Interface and the “Subdural Space”

Under physiological conditions, the dura mater, the dural border cell (DBC) layer, and the arachnoid barrier cell layer are closely apposed such that no true subdural space exists. The DBC layer consists of flattened fibroblast-like cells embedded in a sparse extracellular matrix with weak intercellular junctions, making this interface structurally fragile and susceptible to cleavage [44,45,46,47]. When fluid accumulates along this plane of least resistance, separation of the DBC layer occurs, creating a potential space that may subsequently fill with blood or other fluids and form a subdural collection [46,48].

This layer is particularly prone to mechanical disruption because it contains relatively little extracellular collagen and weak cellular connections. Consequently, it represents the anatomical site where CSDHs are believed to originate [12].

### 6.2. Initiating Events and the Role of Trauma

Traditionally, CSDH was attributed to rupture of bridging veins as they traverse the DBC layer. According to this classical theory, traumatic brain injury results in venous bleeding into the subdural space, initially producing an acute subdural hematoma (ASDH) that gradually evolves into a chronic collection over time [17].

However, several observations challenge this concept. Selective rupture of bridging veins without concomitant hemorrhage into the subarachnoid space appears unlikely, the distribution of blood in CSDH often extends across the cerebral convexities rather than near the venous sinuses, and symptoms typically develop weeks after trauma rather than within days [46,49]. Moreover, autopsy studies rarely demonstrate torn bridging veins, and many patients with normal initial CT scans develop CSDH weeks or months later [49].

Current evidence suggests that traumatic events—often minor—may instead damage the DBC layer or the delicate capillary plexus of the inner dura. This plexus, derived largely from the MMA, lies within 5–15 μm of the DBC layer and represents a plausible source of bleeding into the subdural compartment [49].

In addition to trauma, other factors such as cerebral atrophy in elderly patients, anticoagulation therapy, intracranial hypotension, or prior surgery may predispose the DBC layer to disruption and subsequent fluid accumulation [50,51,52].

### 6.3. Inflammation and Neomembrane Formation

Disruption of the DBC layer initiates a foreign-body–like inflammatory response characterized by macrophage recruitment, granulation tissue formation, and fibroblast activation [46,53,54,55]. Persistent inflammatory activation leads to the formation of vascularized membranes surrounding the hematoma cavity [46,49].

These membranes consist of two distinct layers. The inner membrane, located adjacent to the arachnoid, contains collagen and fibroblasts and is relatively avascular and biologically inactive. In contrast, the outer membrane originates from the dura mater and represents the primary source of repeated bleeding in CSDH [56].

Histologically, outer membranes have been classified into four types according to their inflammatory activity and maturity [5,57]:

- Type I: non-inflammatory membrane with immature fibroblasts and minimal neovascularization.
- Type II: inflammatory membrane with significant cellular infiltration and angiogenesis.
- Type III: hemorrhagic-inflammatory membrane with multiple fragile vessels and active bleeding.
- Type IV: scar-inflammatory membrane characterized by fibrosis with residual inflammatory activity.

The newly formed capillaries within these membranes are structurally immature and fragile, making them prone to recurrent microhemorrhages that sustain hematoma growth. This chronic inflammatory process therefore plays a central role in the persistence and progression of CSDH [3,46].

### 6.4. Angiogenesis and the Role of the MMA

Recent histological and angiographic studies have demonstrated that the neovasculature within the outer membrane is supplied predominantly by distal branches of the MMA. These small arterial branches penetrate the dura and feed fragile capillaries within the subdural membranes [56,58].

This vascular supply explains why embolization of the MMA can effectively reduce recurrent bleeding and inhibit further hematoma expansion by devascularizing the pathological neomembranes [59].

Several angiogenic mediators contribute to this pathological vascularization. Vascular endothelial growth factor (VEGF) is markedly elevated in hematoma fluid—often exceeding serum levels by more than twenty-fold and promotes endothelial proliferation and increased vascular permeability [60]. The angiopoietin-2 (Ang-2)/Tie2 pathway further destabilizes vascular structures, while matrix metalloproteinases (MMP-1, MMP-2, and MMP-9) degrade extracellular matrix and endothelial junction proteins, facilitating angiogenesis but rendering newly formed vessels structurally fragile [61,62,63].

Additional inflammatory mediators, including interleukins (IL-6, IL-8, and IL-1β) and tumour necrosis factor-α (TNF-α), promote leukocyte recruitment, endothelial permeability, and sustained inflammatory activation within the hematoma cavity [46]. Oxidative stress from erythrocyte breakdown releases heme and iron, further amplifying inflammatory signaling and membrane formation [64].

### 6.5. Mechanisms of Hematoma Persistence and Expansion

CSDH progression is driven by a combination of persistent inflammation, pathological angiogenesis, and biochemical imbalance within the hematoma cavity. Elevated fibrinolytic activity, including increased levels of tissue plasminogen activator (tPA) and urokinase plasminogen activator (uPA), prevents stable clot formation and contributes to continuous fluid accumulation [65].

Neocapillaries within the outer membrane are highly permeable and continuously leak protein-rich fluid into the subdural space. Meanwhile, the inner membrane acts as a semi-permeable barrier that impedes hematoma resorption [66].

**Figure 4:**
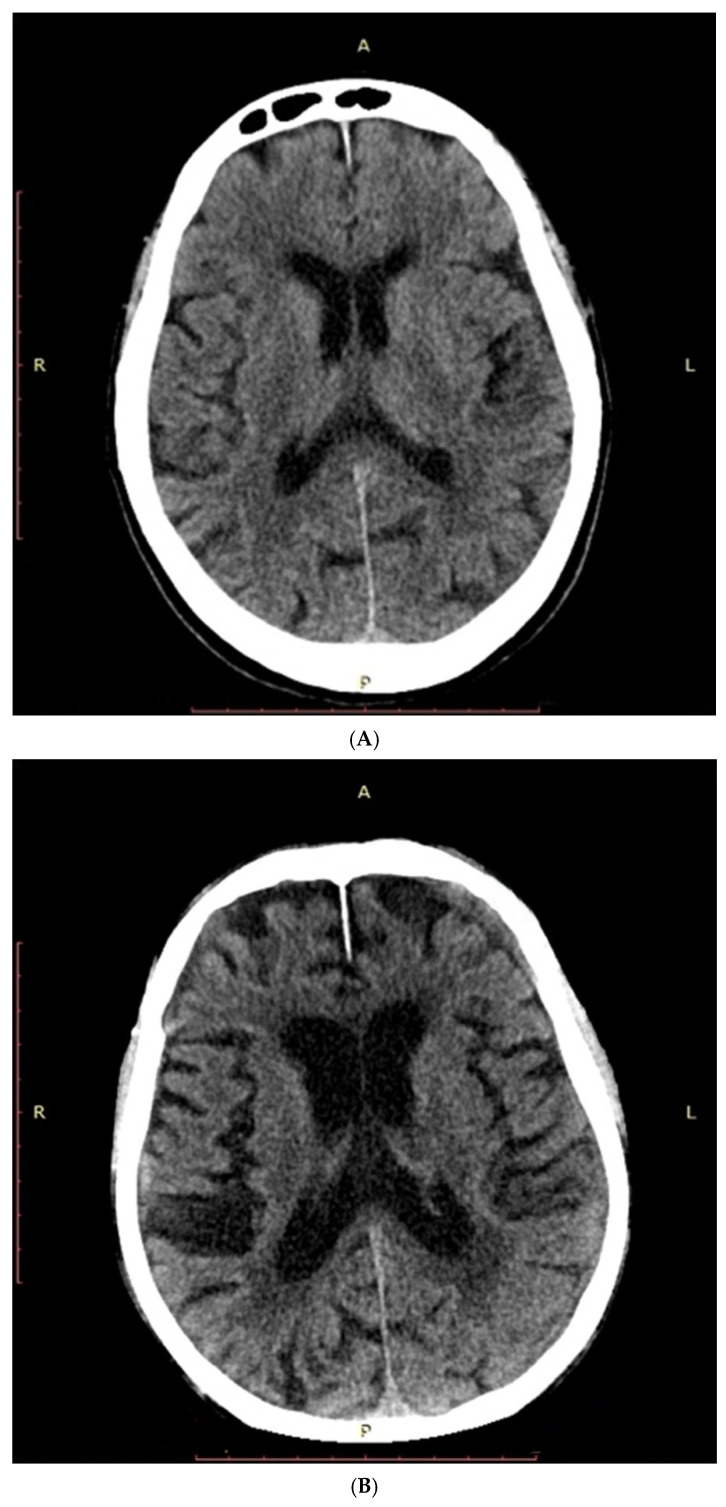
(**A**) Axial section of the brain on non-contrast CT (radiological image of a normal distribution of brain tissue). (**B**) Axial section of the brain on non-contrast CT (radiological image of cortical–subcortical atrophy with enlargement of intracranial fluid spaces, typical of brain atrophy). Own material. A—Anterior; L—Left; P—Posterior; R—Right.

Mechanical factors also contribute to hematoma persistence. In elderly individuals with cerebral atrophy (Figure 4), enlargement of the subdural compartment allows gradual expansion of the collection without substantial elevation in intracranial pressure [48].

Some investigators have additionally proposed that CSDH may develop following subdural hygromas. In this model, cerebrospinal fluid accumulation within the dural border layer creates a potential space that predisposes to subsequent bleeding and hematoma formation [67,68,69].

### 6.6. Temporal Evolution of CSDH

The development of CSDH can be broadly divided into three stages: initial formation, latency, and clinical manifestation. During the initial phase, minor trauma damages dural capillaries or the DBC layer, resulting in a small hemorrhage that may not be detectable on early CT imaging [3].

During the latency period, progressive neomembrane formation and recurrent microhemorrhages lead to gradual enlargement of the hematoma cavity, often while patients remain asymptomatic. Finally, weeks after the initial injury, progressive expansion of the hematoma capsule results in increased intracranial pressure and neurological symptoms such as headache, seizures, or decreased consciousness [3,17].

### 6.7. Therapeutic Implications

Recognition of the central role of pathological neovascularization and MMA-derived blood supply in CSDH has important therapeutic implications. Treatments that target the vascular supply of the subdural membranes—most notably MMA embolization—aim to interrupt this pathological cycle of inflammation, angiogenesis, and recurrent bleeding [70,71,72,73].

## 7. Clinical Presentation of CSDH

### 7.1. Spectrum of Clinical Manifestations

CSDH presents with a wide spectrum of neurological manifestations ranging from completely asymptomatic cases to severe neurological impairment and loss of consciousness [5,11,74]. Symptoms often develop insidiously and may be delayed for weeks or even months following the inciting event, which is frequently minor or unrecognized trauma [75]. Because of this variability and the diversity of symptoms, CSDH has often been described as *“the great neurological imitator”* [76].

The most commonly reported symptoms include headache, confusion, cognitive decline, gait disturbance, focal neurological deficits, seizures, and limb weakness [3,11,77]. In elderly patients, altered mental status is particularly frequent, occurring in approximately 50–70% of cases [78,79]. Headache and confusion are among the most prevalent presenting complaints [1]. Other neurological manifestations may include aphasia, numbness, drowsiness, ataxia, dysphagia, and difficulties with speech or ambulation [74]. Less frequently, patients may present with seizures, which occur in up to 6% of cases as an initial symptom [80]. In individuals with preexisting epilepsy, an increase in seizure frequency may signal the development of CSDH [81].

Focal neurological deficits are also common. Hemiparesis or hemisensory deficits are often contralateral to the hematoma due to direct compression of the cerebral hemisphere [80]. In one clinical series, hemiparesis was observed in 58% of patients [80]. Additional focal manifestations may include transient neurological deficits, language disturbances, or isolated cranial nerve palsies [75]. Rare presentations such as vertigo, nystagmus, oculomotor palsy, and even Parkinsonian symptoms have also been reported, likely related to compression of the basal ganglia, midbrain structures, or cranial nerves secondary to mass effect [82,83].

Large clinical cohorts have reported that focal neurological deficits occur in approximately 46% of patients, headache in 41%, gait disturbance in 31%, and cognitive impairment in 31% [1]. Importantly, cognitive decline and reduced consciousness have been associated with poorer short-term functional outcomes, whereas headache at presentation may be associated with more favorable outcomes [1]. Table 2 summarizes the common clinical manifestations of CSDH.

### 7.2. Neurological Severity and Clinical Grading

The severity of symptoms in CSDH is influenced by several factors, including hematoma volume, rate of hematoma expansion, anatomical location, and the presence of mass effect on the brain parenchyma [11]. Mass effect may manifest radiologically as effacement of cortical gyri, ventricular compression, or signs of cerebral herniation [11].

The level of consciousness is frequently assessed using the Glasgow Coma Scale (GCS). Most patients with CSDH present with relatively preserved consciousness, typically with a GCS score between 13 and 15. Approximately 8% of patients present with moderate impairment (GCS 9–12), while only about 3% present with severe impairment (GCS 3–8) [6,84].

Clinical severity can also be categorized using the Markwalder grading system (Table 3), which classifies patients from grade 0 to grade 4 according to neurological status and symptom severity [85]. This grading system is commonly used in both clinical practice and research to standardize the assessment of neurological impairment in patients with CSDH [11].

### 7.3. Age-Related Differences in Presentation

The clinical presentation of CSDH may differ depending on patient age. Younger patients, typically those under 50 years of age, more commonly present with symptoms related to increased intracranial pressure such as headache, nausea, and vomiting. In contrast, older patients more frequently exhibit focal neurological deficits and cognitive impairment [86]. This difference likely reflects age-related brain atrophy, which allows hematomas to enlarge before producing symptoms but predisposes elderly individuals to neurological deficits once mass effect develops [87]. Table 4 summarizes the factors influencing the severity of symptoms in CSDH.

### 7.4. Natural History and Pathophysiological Evolution

The clinical course of CSDH evolves gradually and can be divided into several stages (Table 5) reflecting the underlying pathophysiological processes. One proposed model describes three major phases. The first phase involves a subclinical traumatic event that disrupts the DBC layer, potentially initiating hematoma formation. This is followed by a second phase characterized by slow hematoma maturation, progressive enlargement, and development of neomembranes over a period of weeks to months. Finally, progressive expansion of the hematoma leads to decompensation of intracranial compensatory mechanisms, resulting in symptoms of cerebral irritation and increased intracranial pressure [3].

Another model describes the evolution of CSDH beginning with a subdural hygroma, in which cerebrospinal fluid accumulates in the subdural space. Approximately 25% of hygromas may progress to CSDH [88]. As the condition evolves, outer and inner membranes develop during the homogeneous stage, often accompanied by recurrent microhemorrhages from fragile neovasculature. The laminar stage, a subtype of this phase, is characterized by increased vascularity and may be associated with higher recurrence rates [89].

Subsequently, the separated stage develops, during which the hematoma divides into multiple layers and expands further, increasing intracranial pressure and susceptibility to rebleeding. An intermediate gradation stage may occur, in which minor head movements can cause mixing of hematoma layers. The final trabecular stage represents a phase of gradual resolution, where fibrous septations form and the hematoma volume slowly decreases, reducing the likelihood of further bleeding. At this stage, symptoms are often related primarily to the mass effect of the hematoma rather than active bleeding [89].

### 7.5. Atypical Presentations and Special Etiologies

Although trauma is the most common cause of CSDH, cases associated with cerebrospinal fluid leakage may present differently. In patients with intracranial hypotension due to dural leaks, symptoms may not appear immediately and often reflect reduced intracranial pressure rather than mass effect [11,90].

A systematic review of intracranial subdural hematomas associated with neuraxial anesthesia found that the most common symptoms were non-postural headaches (81%) and postural headaches (77%) [90]. In addition, tinnitus has been reported in patients with intracranial hypotension, although it is relatively uncommon in CSDH of other etiologies [91].

### 7.6. Clinical Implications for Management

The severity of neurological symptoms often guides treatment decisions. Patients presenting with significant neurological deficits or decreased consciousness typically require urgent surgical intervention to prevent further neurological deterioration [92]. Conversely, asymptomatic or mildly symptomatic patients may be managed conservatively with close observation, particularly if they have substantial surgical risk due to comorbidities. Approximately 40% of hematomas may resolve spontaneously, although about 20% of patients initially treated conservatively eventually require surgical evacuation [93].

## 8. Diagnosis of CSDH

### 8.1. Clinical Evaluation and Diagnostic Challenges

The diagnosis of CSDH begins with a thorough clinical assessment, including detailed history-taking and neurological examination. Important risk factors include prior head trauma, antithrombotic therapy, alcohol abuse, and preexisting neurological disorders [5]. Notably, many patients do not recall recent trauma, as the inciting event may have occurred weeks to months earlier or gone unnoticed [17].

Neurological evaluation focuses on cognitive impairment, altered mental status, focal deficits, and signs of increased intracranial pressure [5]. However, clinical diagnosis remains challenging due to the heterogeneity of symptoms and the limited practical value of time-based definitions of chronicity.

Radiologically, CSDH demonstrates considerable variability in location (convexity, interhemispheric fissure, posterior fossa, or rarely skull base), density (hypodense to isodense), and internal architecture (homogeneous to loculated) [94]. Consequently, no universally accepted diagnostic criteria exist. Although CSDH is commonly defined as a hypo- or isodense subdural collection [95,96], this definition is limited by difficulties in distinguishing it from subdural hygromas [88].

### 8.2. Noncontrast Computed Tomography: First-Line Imaging

Noncontrast CT remains the primary imaging modality for diagnosing and monitoring CSDH. It reliably identifies the presence, size, and location of hematomas, which typically appear as crescent-shaped, hypodense extra-axial collections crossing suture lines [5].

**Figure 5:**
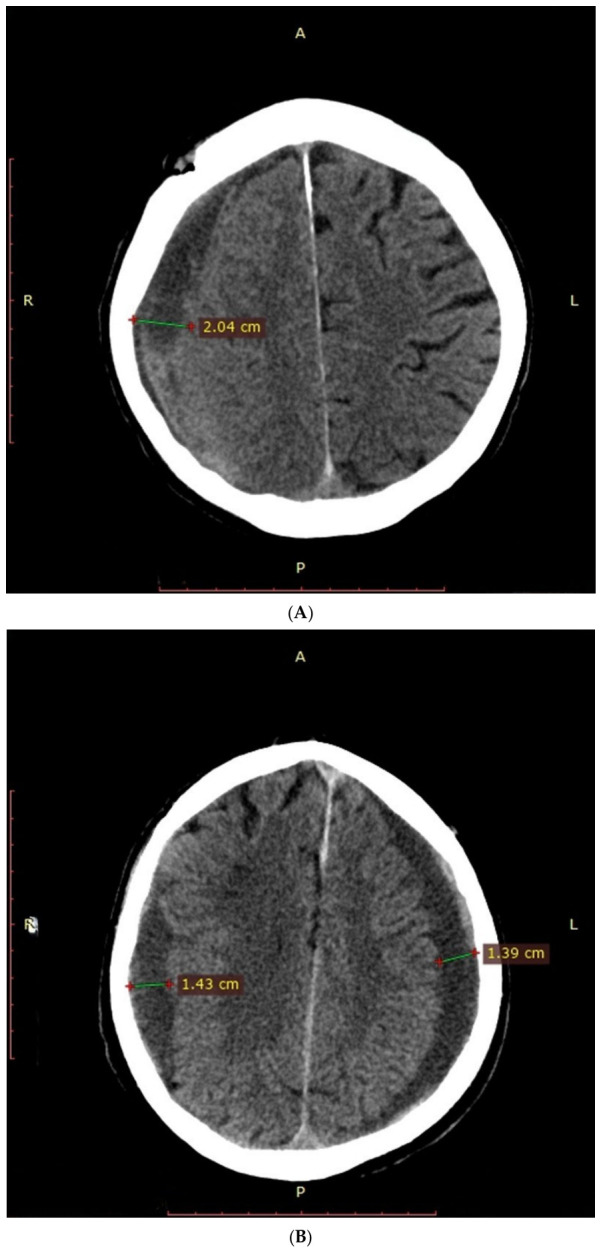
(**A**) Axial section of the brain on non-contrast CT (radiological image of a right-sided subdural hematoma causing marked compression of the brain tissue—measurement of the hematoma width). (**B**) Axial section of the brain on non-contrast CT (radiological image of bilateral subdural hematomas causing marked compression of the brain tissue on both sides—measurement of the hematoma widths). Own material. A—Anterior; L—Left; P—Posterior; R—Right.

CSDH most commonly occurs along the cerebral convexities and may be unilateral or bilateral, with bilateral cases reported in 9–22% of patients (Figure 5) [6,97,98,99,100,101,102]. A slight predominance of left-sided hematomas has been described, possibly due to hemispheric dominance [102].

#### 8.2.1. Key CT Features

CT assessment typically includes: hematoma thickness and extent, midline shift, presence of membranes, mass effect (sulcal effacement, ventricular compression, herniation signs) [17,103]. Midline shift is measured relative to midline structures such as the septum pellucidum, while hematoma thickness is usually assessed on axial or coronal reconstructions [5,17]. A thickness > 10 mm or midline shift > 5 mm often guides surgical decision-making [5], although clinical symptoms remain more important than imaging alone [17]. Mixed-density hematomas may indicate recurrent bleeding [104,105]. Additional findings include hematocrit levels, cortical compression, and ventricular collapse [17,103].

#### 8.2.2. Role in MMA Embolization Planning

In the context of MMA embolization, CT is essential for evaluating: non-emergent mass effect, hematoma thickness, internal structure and membrane presence. Patients considered for embolization typically demonstrate moderate mass effect without herniation, hematoma thickness ≥ 10 mm (institution-dependent), and imaging features suggesting chronicity or recurrence [17].

Imaging findings such as the presence of vascularized membranes and signs of ongoing inflammation or neovascularization are increasingly recognized as important factors in selecting patients for MMA embolization, as these features are associated with hematoma persistence and recurrence [46,106].

### 8.3. Contrast-Enhanced Magnetic Resonance Imaging

**Figure 6:**
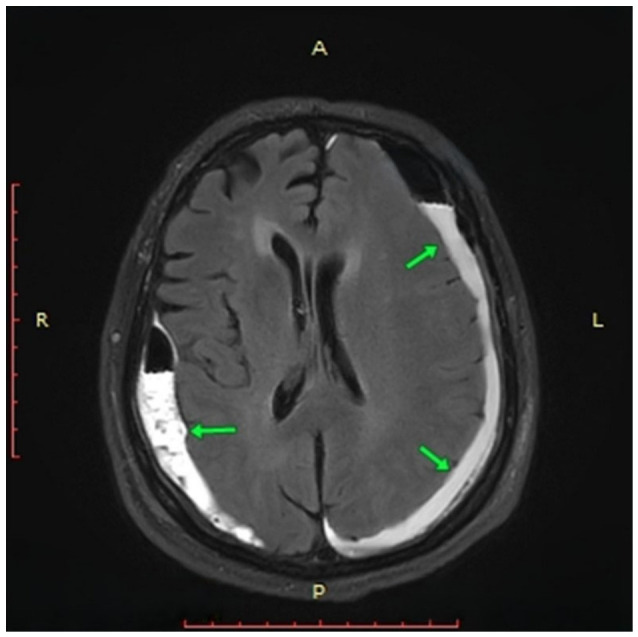
Axial section of the brain in T2 FLAIR sequence on 3T MRI (radiological image of hyperintense bilateral chronic subdural hematomas indicated by arrows). Own material. A—Anterior; L—Left; P—Posterior; R—Right.

Magnetic Resonance Imaging (MRI) provides superior soft tissue characterization and is particularly valuable for assessing hematoma membranes [107]. It enables visualization of internal and external membranes, differentiation between liquid and solid components, and detection of CT-isodense hematomas [108]. Figure 6 shows CSDH on MRI.

Spreer et al. [107] reported visualization of external membranes in 94% of cases (16/17), while internal membranes were primarily seen in later stages. MRI also identifies “spandrel-like” membrane thickening, corresponding histologically to areas of neovascularization [109,110]. This information is clinically relevant, as incomplete membrane removal is associated with recurrence [111], and MRI may guide the choice between burr-hole drainage and craniotomy [108].

### 8.4. Dual-Energy CT

Dual-energy CT (DECT) is an emerging modality that improves membrane visualization and hematoma characterization [112]. Using iodine mapping, DECT can distinguish enhancing membranes from surrounding tissues [113].

Membrane grading on DECT includes [112]:

- Grade I: external membrane only.
- Grade II: early internal membrane (“spandrel sign”).
- Grade III: fully developed internal and external membranes.

Higher grades correlate with increased recurrence risk after surgical evacuation [114,115] and have also been associated with outcomes following MMA embolization [116]. Table 6 compares CT and MRI.

While CT remains the cornerstone of diagnosis, MRI provides complementary information that may be critical in complex or recurrent cases, particularly by identifying membrane characteristics associated with treatment resistance.

### 8.5. Follow-Up Imaging

**Figure 7:**
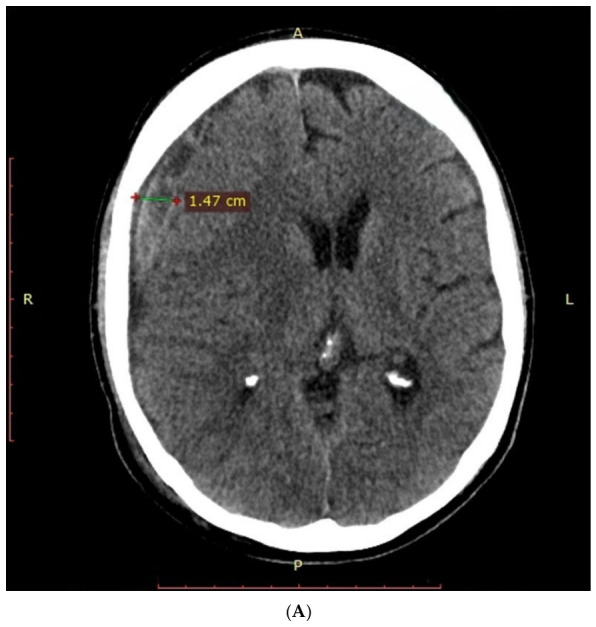

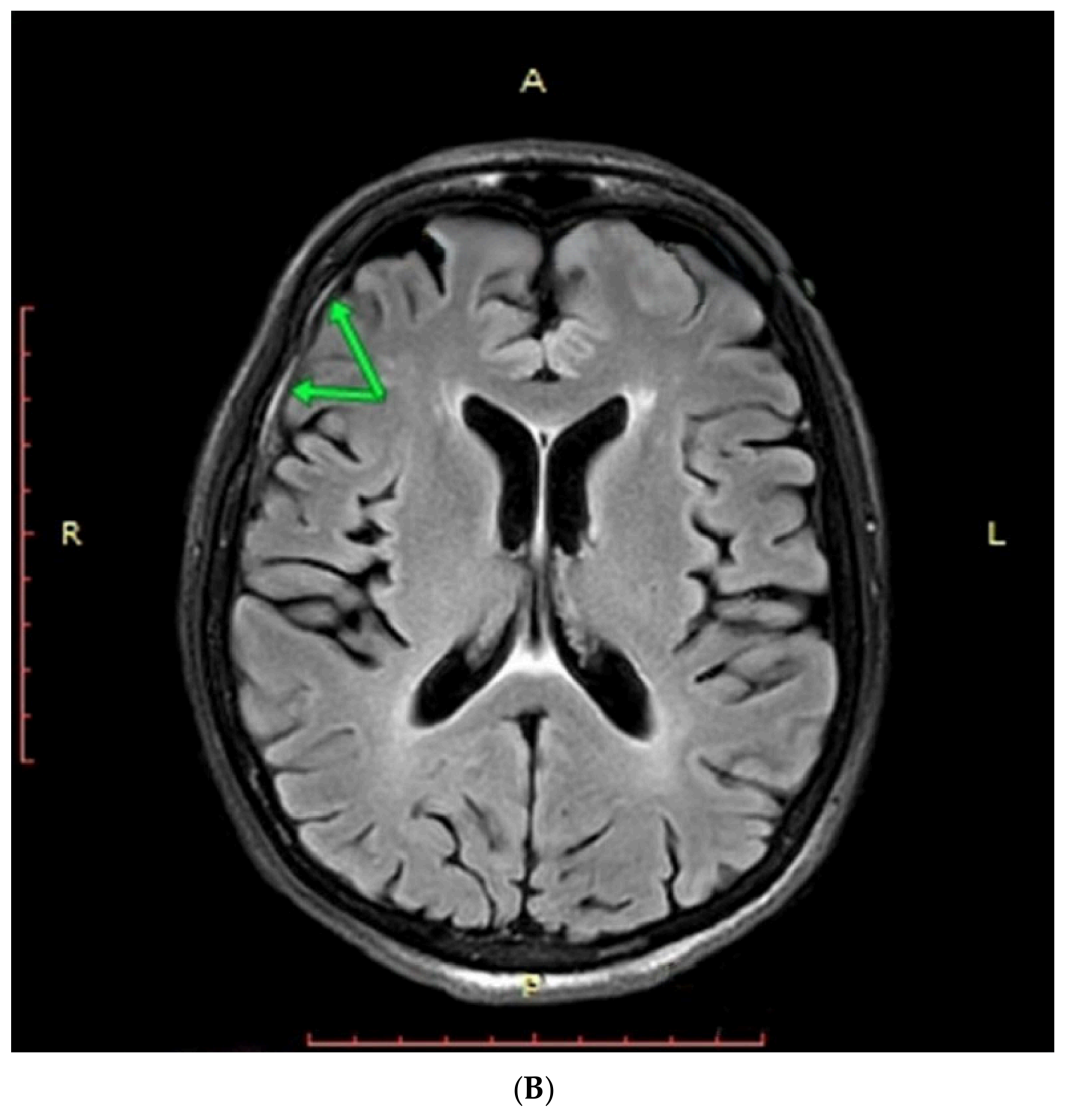
(**A**) Axial section of the brain on non-contrast computed tomography (radiological image of a right-sided subdural hematoma causing marked compression of the brain tissue—measurement of the hematoma width). (**B**) Axial section of the brain in T2 FLAIR sequence on 3T MRI (radiological image, follow-up after embolization with regression of the right-sided subdural hematoma—the hematoma bed is indicated by arrows). Own material. A—Anterior; L—Left; P—Posterior; R—Right.

There is no consensus regarding optimal follow-up imaging protocols after treatment (surgical or MMA embolization). Common practice includes CT imaging at: 1 day, 1 month, 3 months, and 6 months post-procedure [5]. However, recent studies suggest that routine follow-up imaging may have limited clinical impact, as symptomatic recurrence typically precedes scheduled imaging [117,118,119]. For clinically stable patients, imaging may be performed selectively based on symptom progression [120]. Some authors suggest that follow-up strategies should be individualized based on clinical status and treatment modality, with closer monitoring in patients undergoing MMA embolization due to variable rates of hematoma resorption [106]. Figure 7 shows CSDH on CT and follow-up on MRI after MMA embolization.

Based on currently available evidence, a pragmatic follow-up approach may include early postoperative CT imaging (within 24–72 h) after surgical evacuation to assess residual hematoma and complications, followed by additional imaging primarily in symptomatic patients or those at high risk of recurrence. After MMAE, follow-up imaging at approximately 4–6 weeks and again at 3 months may be reasonable to evaluate progressive hematoma resolution, particularly in patients managed conservatively or with persistent residual collections. Nevertheless, these suggestions are not evidence-based guidelines and should be interpreted as proposed clinical considerations requiring prospective validation.

Diagnosis of CSDH relies on a combination of clinical evaluation and imaging, with noncontrast CT as the primary diagnostic tool. Advanced imaging techniques such as MRI and DECT enhance characterization of hematoma membranes, which is particularly relevant for predicting recurrence and guiding treatment strategies, including MMA embolization. Despite advances, standardized diagnostic criteria remain lacking, emphasizing the need for integrated clinical and radiological assessment.

## 9. Treatment Approaches for CSDH

### 9.1. Conventional Surgical Management

For decades, surgical evacuation has remained the reference standard for the treatment of CSDH, particularly in symptomatic patients with significant mass effect. The most commonly employed technique is burr-hole craniotomy with closed-system drainage, which allows effective decompression and is associated with relatively low perioperative morbidity [121,122]. The primary goal of surgery is to alleviate intracranial pressure and reverse neurological deficits caused by hematoma expansion [123].

Despite its effectiveness, recurrence after surgical evacuation remains a major limitation, with an average rate of approximately 10–12% [124,125], although some studies report values around 11% [126]. The use of postoperative drainage has been shown to significantly reduce recurrence rates, as demonstrated in randomized trials such as that by Santarius et al. [127], where recurrence decreased from 24% to 9.3% with drain placement. Recurrence is thought to be driven not by inadequate evacuation but by persistent pathological processes within the hematoma membranes, including inflammation, angiogenesis, and recurrent microhemorrhage [14,51].

Several risk factors for recurrence have been proposed, including advanced age, male sex, anticoagulant or antiplatelet therapy, diabetes mellitus, alcohol use, coagulopathies, and unfavorable hematoma characteristics such as septations or poor brain reexpansion [86,103,128]. However, the relative contribution of these factors remains inconsistent across studies.

Alternative surgical techniques include twist-drill craniostomy, often used in frail patients [129], and mini-craniotomy, which is reserved for organized or septated hematomas and recurrent cases [130]. While more extensive procedures may reduce recurrence in selected patients, they are associated with increased operative time and morbidity [131]. Importantly, all surgical techniques primarily address the mass effect and do not directly target the underlying pathophysiology responsible for recurrence.

### 9.2. Pharmacological Therapies

#### 9.2.1. Statins

Over the past decade, statins have emerged as a potential conservative treatment strategy for CSDH due to their pleiotropic effects on inflammation, angiogenesis, and endothelial stabilization [14]. Preclinical studies demonstrated that statins such as atorvastatin enhance angiogenic maturation, reduce inflammatory cytokines, and promote hematoma resolution [60,132].

Early clinical evidence supports these findings. In a pilot prospective study by Jiang et al. [133], significant hematoma volume reduction and functional improvement were observed in patients treated with atorvastatin. These results were further validated in the ATOCH RCT, which demonstrated that atorvastatin (20 mg daily for 8 weeks) significantly reduced hematoma volume and the need for surgical intervention compared with placebo [13,134]. Neurological outcomes were also improved, with no increase in adverse events [135].

However, more recent evidence has produced less consistent findings. A 2026 propensity score-matched cohort study by Hamou et al. [136] involving 564 surgically treated patients found that the apparent protective association (OR 0.599, *p* = 0.021) between pre-existing statin use and reduced recurrence observed in unadjusted analyses was no longer statistically significant after adjustment for cardiovascular comorbidities and other confounding variables (OR 0.649, *p* = 0.076). The authors concluded that statin therapy was not independently associated with lower postoperative recurrence risk and that hematoma architecture appeared to be a stronger predictor of recurrence.

Interpretation of the available evidence is further limited by heterogeneity in study populations, differences in treatment timing and duration, and variation in surgical versus nonsurgical management strategies. In addition, most studies have focused primarily on radiographic or recurrence endpoints rather than long-term functional outcomes.

Consequently, although statins remain biologically plausible and potentially promising adjunctive therapies, current evidence is insufficient to support their routine use as a standard treatment strategy for CSDH outside selected clinical contexts or ongoing clinical trials.

#### 9.2.2. Tranexamic Acid

Tranexamic acid (TXA), an antifibrinolytic agent, has been investigated as an adjunctive or conservative therapy aimed at stabilizing the hematoma and preventing expansion [137]. Several studies, including a meta-analysis by Musmar et al. [7], reported significantly reduced recurrence rates and hematoma volume with TXA use.

Although TXA shows promise in reducing hematoma expansion, evidence remains limited by study heterogeneity. Meta-analyses such as the one conducted by Pan et al. [138], involving nine studies (five RCTs) reported that TXA significantly lowered recurrence (OR 0.33, 95% CI 0.26–41) and did not increase thrombosis or mortality. However, the same authors cautioned that multicentre RCTs are needed to determine whether TXA improves neurological function or long-term prognosis. Another meta-analyses of 14,836 patients noted that TXA reduced recurrence but evidence remains limited, and heterogeneity among included studies prevents firm conclusions [139].

Importantly, although recurrence reduction appears promising, current evidence has not conclusively demonstrated improvement in long-term neurological recovery, functional independence, or survival. Several authors have therefore emphasized the need for adequately powered multicenter RCTs before TXA can be recommended as an established standard therapy for CSDH.

At present, TXA should be regarded as an investigational or adjunctive therapeutic option with encouraging preliminary data but insufficient high-quality evidence to support universal routine use.

#### 9.2.3. Corticosteroids

Corticosteroids, particularly dexamethasone, have been used to suppress inflammation and angiogenesis within the hematoma membrane [13]. While early observational studies suggested reduced recurrence rates [140,141], more robust evidence has raised concerns. The DEX-CSDH randomized trial demonstrated fewer reoperations but worse functional outcomes and a higher rate of serious adverse events, including hyperglycemia, infection, and psychosis [96]. Meta-analyses have also indicated increased morbidity and possibly mortality associated with steroid use [142,143]. As a result, routine corticosteroid therapy is no longer recommended and is generally reserved for selected cases.

### 9.3. Middle Meningeal Artery Embolization in Chronic Subdural Hematoma

#### 9.3.1. Pathophysiological Rationale and Mechanism of Action

The MMA, a branch of the ECA, supplies the dura mater and plays a critical role in the vascularization of the outer neomembrane in CSDH. MMAE is an endovascular technique in which embolic material is delivered via a microcatheter to occlude distal MMA branches supplying this pathological membrane [18,144,145]. By targeting fragile neovasculature responsible for recurrent microhemorrhages and exudation, MMAE aims to “devascularize” the hematoma membrane, thereby interrupting the cycle of hematoma persistence and recurrence [146].

This mechanism distinguishes MMAE from conventional surgical evacuation, which removes the hematoma but does not directly address the underlying pathophysiological driver. As a result, MMAE has been proposed both as an adjunct to surgery and as a minimally invasive alternative in selected patients [70,147], particularly those with high surgical risk or recurrent disease [148].

#### 9.3.2. Evidence from Observational Studies

A substantial body of observational data supports the effectiveness of MMAE in reducing recurrence and need for reoperation. Since its first description by Mandai et al. [149] in 2000, multiple retrospective and multicenter studies have demonstrated favorable outcomes. In a multicenter study by Kan et al. [125] including 154 patients, MMAE resulted in a low rate of rescue surgery (6.5%) and radiographic improvement in 90.9% of cases. Similarly, Salih et al. [150] reported a significantly lower recurrence rate in patients treated with adjunctive MMAE compared with surgery alone (7.7% vs. 30.8%). Meta-analyses further reinforce these findings. Jumah et al. [151] reported treatment failure rates of 2.8%, complication rates of 1.2%, and surgical rescue rates of 2.7%. Another large meta-analysis by Ironside et al. [144] demonstrated lower surgical rescue rates (4.4% vs. 16.4%) and fewer in-hospital complications (1.7% vs. 4.9%) compared with conventional treatment. Additionally, Ban et al. [146] showed dramatically reduced treatment failure rates when MMAE was used both in nonsurgical and surgical cohorts. Overall mortality associated with MMAE ranges from 0% to 7%, with most studies reporting rates below 5%, and deaths are typically related to underlying comorbidities rather than the procedure itself [144,152,153].

#### 9.3.3. Evidence from Randomized Controlled Trials

Although several recent RCTs have reported favorable effects of MMAE on radiographic recurrence and reoperation rates, the overall evidence remains heterogeneous and interpretation requires caution.

The EMBOLISE trial demonstrated that adjunctive MMAE significantly reduced the need for repeat surgery compared with surgery alone (4.1% vs. 11.3%), without increasing neurological complications. Although mortality at 90 days was slightly higher in the MMAE group (5.1% vs. 3.0%), this difference was not attributed to the procedure or embolic agents [126].

Similarly, the STEM trial showed a substantial reduction in treatment failure at 180 days (16% vs. 36%) with adjunctive MMAE, with no increase in disabling stroke or short-term mortality [154].

The MAGIC-MT trial reported lower rates of recurrence or progression (6.7% vs. 9.9%) and fewer serious adverse events in the MMAE group, although differences in primary endpoints were not statistically significant [155].

More recently, the EMPROTECT trial evaluated MMAE as an adjunct to surgery and reported a lower 6-month recurrence rate in the embolization group compared with controls (14.8% vs. 21.0%), although this difference did not reach statistical significance. Repeat surgery for recurrence was also less frequent in the MMAE group (4.3% vs. 8.3%), with no significant differences in functional outcomes, mortality, or hospital stay. Procedure-related complications were rare, including one major event (0.6%, ischemic stroke without lasting severe deficit) and three minor transient complications (1.8%) [156].

Recent meta-analyses of randomized trials have provided a more nuanced interpretation of these findings. Gillespie et al. [9] analyzed data from the MAGIC-MT, EMBOLISE, and STEM trials and found that MMAE reduced symptomatic progression or recurrence overall, although the result narrowly missed statistical significance (risk ratio (RR) 0.50, 95% CI 0.23–1.06; *p* = 0.058). Importantly, when analysis was restricted to surgically treated patients, MMAE was not associated with significant reductions in recurrence (RR 0.60, 95% CI 0.19–1.88, *p* = 0.194) or improvements in functional outcomes (RR 1.01, 95% CI 0.97–1.04).

Similarly, Jayakumar et al. [157], in a meta-analysis including four RCTs (EMBOLISE, STEM, MAGIC-MT and EMPROTECT), confirmed a reduction in recurrence requiring surgery (pooled RR 0.40, 95% CI 0.28–0.58) but highlighted a number needed to treat of 15. Crucially, this study found no benefit in mortality (RR 0.94, *p* = 0.85) or functional independence, and a UK cost analysis suggested that universal MMAE implementation could result in a net financial loss of £1.6–1.9 million under current National Health Service tariffs. Therefore, the therapeutic value of MMAE may be localized primarily to non-surgical candidates, and its clinical adoption must balance procedural costs against marginal benefits.

These findings suggest that the principal benefit of MMAE may relate primarily to reduction in radiographic or surgical recurrence rather than improvement in broader patient-centered outcomes such as neurological recovery, functional independence, or survival. Moreover, the magnitude of benefit appears to vary according to patient selection, treatment strategy (adjunctive versus primary embolization), and hematoma characteristics.

Interpretation of the current evidence is additionally limited by heterogeneity in trial design, embolic materials, follow-up duration, outcome definitions, and crossover rates. Several trials were also industry-sponsored, which should be considered when interpreting efficacy estimates.

Consequently, although MMAE appears promising as an adjunctive strategy in selected patients with CSDH, current randomized evidence does not yet establish MMAE as a universal standard of care.

#### 9.3.4. Technical Considerations and Procedural Workflow

MMAE is performed using endovascular techniques, increasingly via transradial access due to its lower complication rates and improved tolerability in elderly patients with complex vascular anatomy [148,158]. Radial access is associated with reduced bleeding complications, shorter immobilization time, and shorter hospital stays compared with femoral access [158,159].

The procedure involves catheter navigation from the radial artery through the brachial and subclavian arteries to the ECA, followed by selective catheterization of the MMA [17]. Superselective angiography is performed to map vascular anatomy and identify dangerous anastomoses, particularly with the OphA [125].

A microcatheter is advanced distally within the MMA to minimize the risk of non-target embolization. Once optimal positioning is achieved, embolic material is injected under fluoroscopic guidance. Adjunctive measures such as intra-arterial lidocaine may be used to improve patient comfort and reduce vasospasm [160].

**Figure 8:**
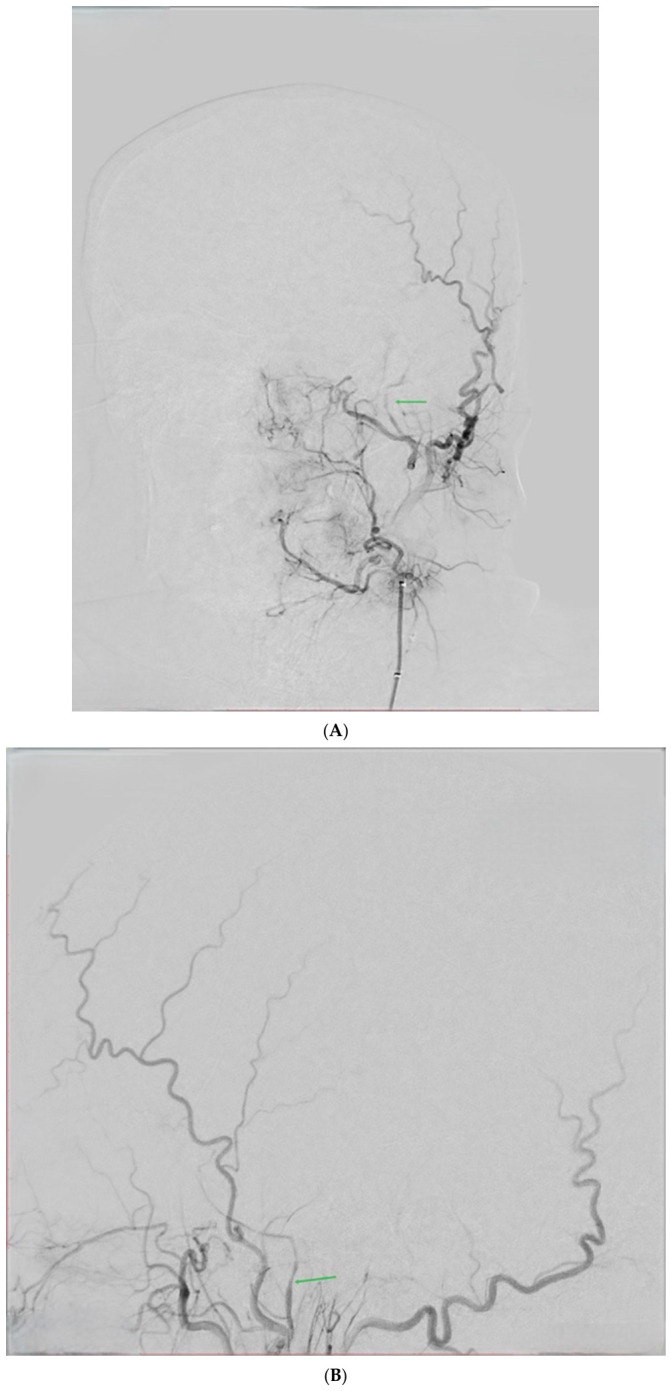
Anterior–posterior (**A**) and lateral (**B**) projections of angiography of the left ECA after selective embolization of the MMA (the course of the trunk of the MMA is indicated by the green arrow). Own material.

Postprocedural imaging is performed to confirm embolization success and exclude complications (Figure 8) [17].

#### 9.3.5. Embolic Agents

**Figure 9:**
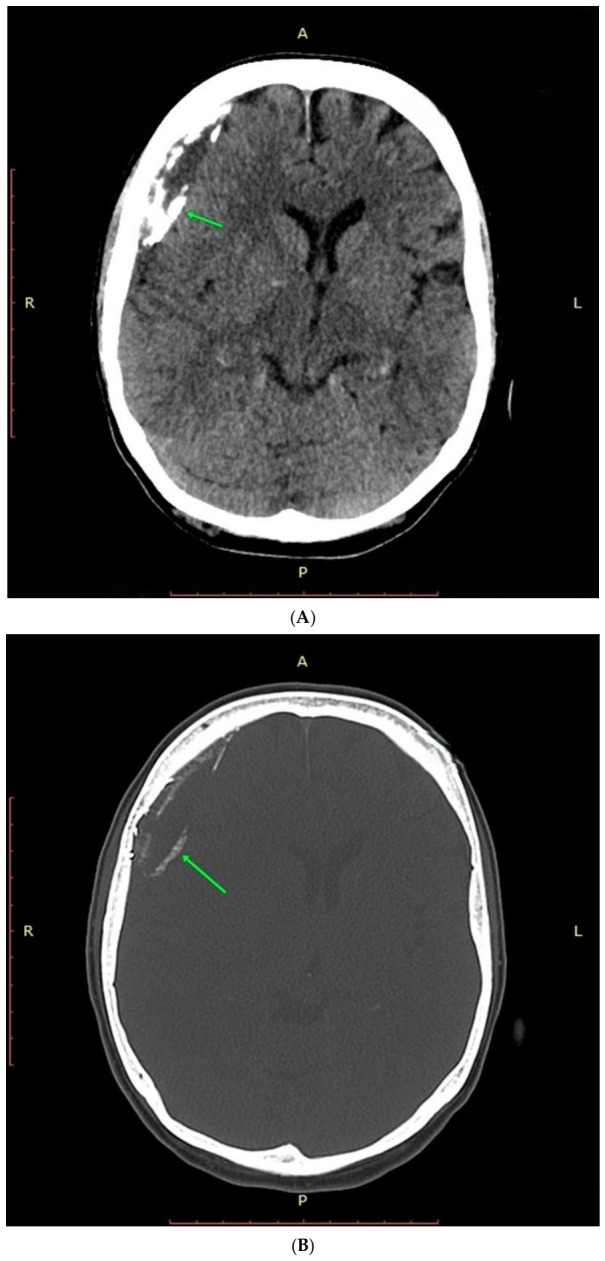
Axial section of the brain on non-contrast CT in both the standard window (**A**) and bone window (**B**) (within the hematoma capsule, a hyperdense focus is visible, indicated by an arrow, corresponding to deposited embolic material—20% histoacryl glue [n-butyl cyanoacrylate, n-BCA]). Own material. A—Anterior; L—Left; P—Posterior; R—Right.

A variety of embolic materials are used in MMAE, each with distinct advantages and limitations. Particle agents such as polyvinyl alcohol (PVA) and microspheres occlude vessels mechanically and induce inflammatory changes but may aggregate and lead to proximal occlusion [160]. Liquid embolic agents, including ethylene vinyl alcohol copolymers (e.g., Onyx, Squid, PHIL) and n-butyl cyanoacrylate (n-BCA) (Figure 9), allow for deeper distal penetration and more durable occlusion of neovasculature [161,162].

Comparative studies suggest similar clinical outcomes between particle and liquid embolics in terms of hematoma reduction and recurrence rates, although liquid agents may provide more homogeneous distribution and better distal penetration [125]. Coils are primarily used for proximal occlusion and are often combined with other agents, as they do not effectively penetrate distal vascular networks [163]. To date, no randomized trials have definitively established the superiority of any specific embolic agent.

#### 9.3.6. Safety Profile and Complications

MMAE has a favorable safety profile, with overall complication rates of approximately 3% and major adverse events occurring in less than 1% of cases [164]. Reported complications include ischemic stroke, cranial nerve palsy, and scalp necrosis, all of which are rare [115].

One of the most serious risks is inadvertent embolization of OphA anastomoses, which can result in irreversible visual loss. This underscores the importance of meticulous angiographic assessment and distal microcatheter positioning [14]. Other potential complications include facial nerve palsy due to embolization of petrosal branches [165]. Careful technique, including avoidance of reflux and appropriate embolic agent selection, is essential to minimize these risks.

#### 9.3.7. Clinical Indications and Patient Selection

MMAE is most commonly used as an adjunct to surgical evacuation to reduce recurrence risk, particularly in patients with known risk factors such as anticoagulation therapy, bilateral hematomas, or prior recurrence [126]. It is also increasingly considered as a primary treatment in selected patients with mild to moderate symptoms or high surgical risk [166].

However, MMAE is not appropriate in all cases. Patients with acute neurological deterioration, significant mass effect, or acute hemorrhagic components generally require urgent surgical decompression [126]. Current practice remains heterogeneous, and ongoing trials such as the CHESS study are expected to further define optimal patient selection and indications for MMAE [166].

#### 9.3.8. Proposed Clinical Decision Framework

Although growing evidence supports the use of surgical, pharmacological, and endovascular strategies in the management of CSDH, universally accepted treatment algorithms remain lacking. Current therapeutic decisions continue to rely on a combination of clinical presentation, radiological findings, patient comorbidities, recurrence risk, and institutional expertise.

Based on the currently available literature, recent RCTs, and emerging consensus statements, a preliminary evidence-informed clinical decision framework may assist in individualized treatment selection (Table 7). In general, patients presenting with significant neurological deficits, marked mass effect, substantial midline shift, or acute neurological deterioration require urgent surgical evacuation. Conversely, conservative management with clinical and radiological monitoring may be appropriate in carefully selected patients with minimal symptoms, limited mass effect, or elevated surgical risk.

Adjunctive MMAE is increasingly being considered in patients perceived to be at higher risk of recurrence, including those with recurrent or bilateral hematomas, anticoagulant use, poor brain re-expansion, or complex membranous hematoma architecture. In selected high-risk or frail patients, primary MMAE may also represent a potential minimally invasive treatment strategy. However, current evidence remains heterogeneous, and available randomized studies have primarily demonstrated reductions in recurrence-related endpoints rather than consistent improvements in functional outcomes or mortality.

Therefore, the proposed framework should be interpreted as a conceptual summary of evolving evidence rather than a formal clinical guideline. Patient selection criteria, optimal timing of embolization, and the comparative effectiveness of MMAE relative to established surgical techniques remain areas of ongoing investigation.

## 10. Limitations

The current body of literature regarding the management of CSDH features several clinical and methodological limitations, highlighting that much of the available data are not entirely definitive. A significant challenge is the lack of standardization, as there are currently no universally accepted diagnostic criteria for CSDH. Furthermore, no clear consensus or evidence-based guidelines exist regarding optimal follow-up imaging protocols after surgical evacuation or middle MMAE. Available clinical data are also heavily limited by significant heterogeneity across study populations, trial designs, embolic materials used, treatment durations, outcome definitions, and patient crossover rates.

Inconsistencies in pharmacological efficacy further complicate management; although therapies like statins and TXA show biological promise, their standalone efficacy remains variable and controversial. Propensity score-matched data indicate that pre-existing statin use is not independently associated with a lower risk of postoperative recurrence, with hematoma architecture serving as a stronger predictor. Meanwhile, TXA studies suffer from high heterogeneity and have failed to conclusively prove improvements in long-term neurological recovery, functional independence, or survival. Corticosteroids like dexamethasone have even been linked to worse functional outcomes and higher rates of serious adverse events, meaning routine therapy is no longer recommended.

Ambiguities also surround MMAE outcomes. While MMAE effectively minimizes recurrence in observational series, data from RCTs remain mixed, with some studies failing to demonstrate significant benefits on primary clinical endpoints. Crucially, when analyses are restricted strictly to surgically treated patients, MMAE has not demonstrated a statistically significant reduction in recurrence or a definitive improvement in functional outcomes. Additionally, universal implementation of interventional endovascular procedures like MMAE carries substantial financial implications and could result in a net financial loss under certain national healthcare tariffs. Ultimately, across emerging nonsurgical therapies for CSDH, including MMAE, statins, and TXA, reductions in radiographic recurrence do not necessarily translate into improved functional outcomes, quality of life, or mortality, meaning future studies should prioritize patient-centered endpoints in addition to recurrence rates alone.

## 11. Future Directions

To resolve existing ambiguities and advance the clinical care of CSDH, future research must move away from restating known limitations and instead pursue a proactive, structured investigation agenda. Establishing the definitive role of MMAE within standardized treatment algorithms requires precise patient selection, focusing heavily on identifying which specific clinical subgroups derive the greatest benefit from MMAE as a primary treatment versus an adjunctive therapy. Comparative trials are also urgently needed to optimize procedural techniques, directly comparing the technical and clinical effectiveness of different embolic materials—such as liquid embolics, particles, or coils—and refining distal catheter positioning strategies.

Incorporating advanced imaging modalities, specifically contrast-enhanced MRI and DECT, into future trial designs will further refine patient selection by better characterizing hematoma membranes and predicting recurrence risks. Investigators should also explore the potential synergistic outcomes of multimodal treatment regimens, specifically evaluating the integration of MMAE combined alongside targeted pharmacotherapy or standard surgical drainage.

Finally, an important methodological caveat in the current CSDH literature is the prevalence of industry sponsorship. Many of the landmark RCTs evaluating embolic agents or pharmaceutical therapies were funded by manufacturing companies. While financial backing accelerates clinical research, industry sponsorship is statistically correlated with favorable outcomes. Future independent, investigator-initiated trials are crucial to eliminate commercial bias and provide objective comparative-effectiveness data.

## 12. Conclusions

CSDH is a complex and highly dynamic neurosurgical condition. Its progression is fundamentally driven by underlying interconnected networks of chronic inflammation, pathological angiogenesis, and recurrent microhemorrhages within dural neomembranes, rather than a simple mechanical accumulation of fluid.

MMAE represents a promising, minor-interventional approach that directly targets the biological vascular supply feeding these pathological membranes. Accumulating observational data and select randomized controlled trials show that MMAE can successfully lower radiographic recurrence rates and reduce the necessity for subsequent rescue operations, particularly when utilized as an adjunct to conventional burr-hole craniostomy.

Nevertheless, because the wider clinical evidence base remains mixed and highly nuanced, MMAE, statins, and alternative pharmaceutical agents cannot yet be heralded as universal standard-of-care options. To safely optimize long-term patient-centered recovery and establish concrete clinical algorithms, the neurointerventional community must rely on future high-quality, independent prospective trials to deliver definitive answers.

## Figures and Tables

**Table 1:** Major branches of the MMA, their vascular territories, anastomoses, and clinical relevance.

Branch of MMA	Main Territory Supplied	Key Anastomoses	Clinical Relevance
Anterior branch	Dura of the frontal and anterior parietal convexities	Anterior falcine branch of the OphA; lacrimal branch of the OphA via the meningolacrimal artery	Risk of retinal ischemia during embolization due to potential reflux into ophthalmic circulation
Posterior branch	Parietotemporal dura and posterior convexity	May communicate with falcine and posterior meningeal vessels	Frequently targeted during embolization for CSDH because it supplies membranes over the parietal convexity
Petrosal branch	Petrous apex region	Internal auditory artery from the anterior inferior cerebellar artery; gives rise to the superior tympanic branch supplying the facial nerve and geniculate ganglion	Important in procedures near the petrous temporal bone; potential involvement in vascular supply to cranial nerve structures
Petrosquamosal branch	Posterolateral floor of the middle cranial fossa, lateral tentorium, dura of the superior posterior fossa	Jugular branch of the ascending pharyngeal artery, medial and lateral tentorial arteries, mastoid branch of the occipital artery	Provides transosseous collateral supply; relevant in skull-base vascularization
Falcine arteries	Falx cerebri	Anterior falcine artery, ethmoidal branches of the OphA, anterior cerebral artery, posterior meningeal artery	Midline collateral circulation; potential cross-hemispheric supply
Cavernous branch	Lateral wall of the cavernous sinus	Posterior branch of the inferolateral trunk of the internal carotid artery	Potentially hazardous extracranial–intracranial anastomosis; supplies the Meckel (trigeminal) cave

**Table 2:** Common Clinical Manifestations of CSDH.

Symptom Category	Clinical Manifestations	Approximate Frequency/Notes	References
General symptoms	Headache, confusion	Among the most common presenting complaints	[1]
Cognitive symptoms	Cognitive decline, altered mental status	Present in ~50–70% of elderly patients	[78,79]
Motor deficits	Hemiparesis, limb weakness	Hemiparesis reported in up to 58% of cases	[80]
Gait disturbances	Gait instability, impaired ambulation	Occurs in ~31% of patients	[1]
Focal neurological deficits	Hemiparesis, hemisensory deficits, cranial nerve palsies	Often contralateral to hematoma	[75,80]
Language disturbances	Aphasia, speech difficulties	May occur in focal presentations	[74]
Other neurological symptoms	Numbness, ataxia, dysphagia	Variable presentation	[74]
Seizures	New-onset seizures or increased seizure frequency	Initial symptom in up to 6% of cases	[80,81]
Rare manifestations	Vertigo, nystagmus, oculomotor palsy, Parkinsonian symptoms	Rare, related to mass effect	[82,83]

**Table 3:** Markwalder Grading Scale for CSDH (Modified from Markwalder et al. [85]).

Grade	Description
0	Neurologically normal; no clinical symptoms.
1	Alert and oriented; mild symptoms such as headache; no neurological deficit or only minimal deficits (e.g., reflex asymmetry).
2	Drowsy or disoriented with variable neurological deficits, such as hemiparesis.
3	Stuporous but responding appropriately to noxious stimuli; severe focal neurological deficits, such as hemiplegia.
4	Comatose with absent motor responses to painful stimuli; may exhibit decerebrate or decorticate posturing.

**Table 4:** Factors Influencing the Severity of Symptoms in CSDH.

Factor	Clinical Relevance	Reference
Hematoma volume	Larger hematomas are associated with greater neurological impairment	[11]
Rate of hematoma expansion	Rapid expansion may lead to acute neurological deterioration	[11]
Hematoma location	Determines type of focal neurological deficit	[11]
Mass effect	May cause ventricular compression, cortical effacement, or herniation	[11]
Patient age	Older patients more likely to present with neurological deficits	[86,87]
Brain atrophy	Allows larger hematoma volumes before symptoms develop	[87]

**Table 5:** Stages of CSDH Development.

Stage	Pathophysiological Features	Clinical Relevance	References
Subdural hygroma stage	CSF accumulation in subdural space	~25% may progress to CSDH	[88]
Homogeneous stage	Formation of inner and outer membranes; recurrent microhemorrhages	Progressive hematoma maturation	[89]
Laminar stage	Increased vascularity within membranes	Associated with higher recurrence rates	[89]
Separated stage	Hematoma divides into layers	Increased intracranial pressure and rebleeding risk	[89]
Gradation stage	Mixing of hematoma layers with head movement	Transitional stage	[89]
Trabecular stage	Fibrous septa formation and gradual hematoma resolution	Lower bleeding risk	[89]

**Table 6:** CT vs. MRI: Comparative Perspective.

Feature	CT	MRI
Availability	Widely available, rapid	Less available, longer acquisition
First-line modality	Yes	No
Detection of hematoma	Excellent	Excellent
Density/attenuation assessment	Yes	Limited
Membrane visualization	Limited	Superior
Differentiation (solid vs. liquid)	Limited	Excellent
Surgical planning	Basic	Advanced (membrane mapping)
Role in MMA embolization	Primary tool	Adjunct in complex cases

**Table 7:** Proposed preliminary clinical decision framework for the management of CSDH based on currently available evidence.

Clinical Scenario	Typical Features	Preferred Initial Management	Potential Role of MMAE	Comments/Limitations
Asymptomatic or minimally symptomatic CSDH	Markwalder 0–1; minimal midline shift; small hematoma	Conservative observation with serial imaging	May be considered in selected high-risk recurrence patients	Many hematomas resolve spontaneously
Mild symptomatic CSDH	Markwalder 1–2; moderate hematoma size; stable neurological status	Individualized management (conservative vs. burr-hole drainage)	Adjunctive or primary MMAE may be considered in selected patients	Evidence for primary MMAE remains limited
Symptomatic CSDH with significant mass effect	Markwalder 2–3; midline shift >5 mm; hematoma thickness >10 mm	Burr-hole evacuation with drainage	Adjunctive MMAE may reduce recurrence risk	Functional benefit remains uncertain
Recurrent CSDH after surgery	Radiographic reaccumulation and/or recurrent symptoms	Repeat evacuation or multimodal approach	MMAE increasingly considered as adjunctive therapy	Selection criteria remain heterogeneous
High recurrence-risk patients	Bilateral hematomas; anticoagulant therapy; septations; poor brain re-expansion	Surgical management with closer follow-up	Adjunctive MMAE may be reasonable	Evidence strongest for recurrence reduction rather than functional outcomes
Frail or high surgical-risk patients	Advanced age; severe comorbidities; anesthesia risk	Individualized minimally invasive strategy	Primary MMAE may be considered in carefully selected cases	Requires close radiological and clinical monitoring
Acute neurological deterioration	Markwalder 3–4; rapid decline; herniation signs; acute bleeding component	Urgent surgical decompression	MMAE alone not appropriate	Surgery remains first-line treatment

## Data Availability

No new data were created or analyzed in this study.

## Abbreviations

The following abbreviations are used in this manuscript:

aMA	Accessory meningeal artery
Ang-2	Angiopoietin-2
BA	Basilar artery
CSDH	Chronic subdural hematoma
CT	Computed tomography
DBC	Dural border cell
DECT	Dual-energy computed tomography
DSA	Digital subtraction angiography
ECA	External carotid artery
FS	Foramen spinosum
GCS	Glasgow Coma Scale
ICA	Internal carotid artery
IL	Interleukin
MA	Maxillary artery
MMA	Middle meningeal artery
MMAE	Middle meningeal artery embolization
MMP	Matrix metalloproteinases
MRI	Magnetic resonance imaging
n-BCA	n-butyl cyanoacrylate
OphA	Ophthalmic artery
PVA	Polyvinyl alcohol
RCT	Randomized controlled trial
RR	Risk ratio
SA	Stapedial artery
TNF	Tumour necrosis factor
TXA	Tranexamic acid
VEGF	Vascular endothelial growth factor
